# Scarlet Fever

**Published:** 1897-11-06

**Authors:** 


### SCARLET FEVER.

Two communications have recently appeared on the treatment of scarlet fever, which deserve careful attention, however little we may agree with the authors in either case.

The first of these is a paper in the *Lancet* by Mr. Wiglesworth, of Liverpool, advocating the internal use of carbolic acid, both as a means of treatment and of prophylaxis. To this the *Lancet* draws special attention in a strongly depreciatory paragraph, which ends as follows: "Such propositions are entirely contrary to all accepted notions of the spread of infectious fevers; but, as Mr. Wiglesworth has the courage of his opinions, we have thought it right to submit his views to the criticism of the profession." This act of condescension will no doubt be received with all due gratitude by Mr. Wiglesworth, although many people may, perhaps, question whether such an introduction is quite courteous, or even fair. The second is a report by Mr. Monk, medical officer of health for Leicester, on the value of the hot acid bath as a remedy for scarlet fever.

### Treatment by Carbolic Acid.

What Mr. Wiglesworth states is this: That if carbolic acid be given in such quantity as to produce darkening of the urine from the beginning of the attack, or, in his words, in the initial stage, the disease is so modified as to become not only less severe, but less infectious; and that, by giving carbolic acid to other members of the family, they became so protected that, when freely exposed to the infection, they either do not take the disease or take it in a very mild form, and from all this he argues that isolation is inexpedient.

We need hardly point out that it would take a large number of cases to prove some of these propositions, a far larger number than the very small array presented by the author of this paper. As regards the conveyance of the infection to others, the matter is somewhat different, for, if we are to believe that the cases he mentions had not been protected by a previous attack, we do get certain facts which would tend to show either that the treatment adopted did have some restraining influence over the infectivity of the disease, or that this infectivity may be much less active than some people are apt to imagine.

The results of "carbolising" the children in the cases which he mentions were as follow:—

Case 1. Two carbolised children were exposed to infection, and both took the disease.

Case 2. One carbolised child was fully exposed to infection and did not take the disease.

Case 3. Two carbolised children and one uncarbolised child were fully exposed to infection, and all remained free.

Case 4. Five carbolised children exposed to infection, and all remained free.

Case 5. One carbolised child remained free.

Thus of his 11 carbolised patients who were presumed to be otherwise susceptible two took the disease and nine escaped.

If we believed that every child exposed to the infection of scarlet fever would be attacked this, of course, would be a very striking result, but as we do not believe anything of the sort we do not feel inclined to accept it as affording any great support to Mr. Wiglesworth's treatment by carbolic acid. It does, however, afford a very considerable support to the idea that "accepted notions" may not express the whole truth.

Everyone knows that scarlet fever is infectious, and that the official view as to the best way of dealing with it is that somehow or other this infection is to be bottled up. But what everybody does not seem to recognise is that we know nothing at all about the reason why one person catches the disease and another not, why sometimes its prevalence extends and at others diminishes, not only irrespective of but absolutely in a contrary sense to the number of centres of infection; and why, as has been shown by Mr. Shirly Murphy to be the case, at the very times when the infectiveness of the disease is greatest its fatality is least. Mr. Wiglesworth may not have helped us much—we do not, indeed, think that he has—but in view of the quagmire of ignorance which surrounds the whole question of the epidemiology of scarlet fever, we ought to welcome every fact which may in any way tend towards enlightenment. "Accepted notions" are dangerous things when they tend to crystalise upon us and clog inquiry.

### The Hot Acid Bath as a Remedy.

We do not propose at the present moment to discuss in any way the many ethical questions which are suggested by this report. We will content ourselves with saying that it appears to us a very dangerous precedent for a talkative and persistent individual to be allowed so to influence the Sanitary Committee of a large town in regard to a question of medical treatment, a thing quite outside both his and their province, that they in turn should presume to suggest a line of treatment to their medical officer, and actually persuade him to carry it out on the patients committed to his care.

The report deals with 500 cases, of which 249 were treated with baths, and 251 in other ways. The average number of baths given was 11 per patient so treated. The average stay in hospital among the bath treated was 30 days, against 38 days among those treated otherwise; while the death-rate was 4 per cent. in the former and 5·1 in the latter.

The number of cases in which complications occurred was larger among the bath treated than among the others, viz., 65 in the former against 52 in the latter.

The complications were as follows, the figures being percentages of the total numbers treated in each case:—

| | Bath Cases. | Otherwise Treated. |
|---|---|---|
| Nephritis ... ... ... | 6·4 ... | 3·1 |
| Glandular Enlargements ... | 15·2 ... | 11·1 |
| Ear and Nose ... ... | 2·4 ... | 3·5 |
| Rheumatism ... ... ... | 2·0 ... | 1·9 |

The largely increased incidence of nephritis and glandular enlargements among the bath cases is a matter of considerable interest.

Of "return" cases 15 were noted, of which 9 were bath treated and 5 not. The statements on the faith of which the bath treatment was undertaken were, that (*a*) no complications follow, (*b*) no peeling or desquamation occurs, (*c*) the fever is more quickly reduced, (*d*) no infection exists after the first few baths, and consequently the patients need not remain long in bed, but, being non-infectious, can join the rest of the family. All these statements have been proved to be untrue. And in regard to the one point in which the bath treatment seems, from the figures given, to have come out better than the ordinary treatment, viz., the lower death rate, we must remember that although we are told that "at no time were the cases for either method of treatment selected," it is also added "unless the patient was obviously too ill to remove frequently from bed." But surely this gives away the whole case! If the bath cases did not include a fair share of those who were "too ill to remove frequently from bed," the comparative statistics are absolutely worthless. What, then, does this "experiment" show? So far as the particular "acid bath treatment" is concerned there is nothing to show but absolute failure in regard to the absurd pretensions put forward by its author—except, indeed, a long series of cases of nephritis and glandular enlargement which ought never to have occurred. Who is responsible for these complications becomes a matter of no small moment. If this matter were seriously brought forward, we feel perfectly sure that the sanitary committee would at once throw the entire responsibility on the medical officer, and in the face of his published statement that no selection of cases was made for either treatment, we cannot but feel that he might find it difficult to show that he exercised due diligence and skill in the treatment of his patients. As a further warning against medical men allowing themselves to be made the tool of irresponsible faddists, we would point out that no sooner did Mr. Monk issue his unfavourable report than the author and originator of the whole plan turned round and asserted that all the evils which had occurred were the result of "the unscientific methods practised at the Fever Hospital." Such is the reward of complaisance!

### How Shall We Best Utilise Our Fever Hospitals?

Putting treatment on one side there are points in these records which raise questions in regard to the full utilisation of our fever hospitals which are of serious interest. In London alone upwards of £160,000 per annum (not counting capital or interest) is being spent in the maintenance of hospitals for isolating infectious diseases, with the result that we manage to isolate about half the cases of scarlet fever that occur at the present season, leaving the rest to do as best they can and spread infection as they like. The excuse for this condition of affairs is that there are not enough beds. Yet we are told by the officers of the Asylums Board that the average stay of patients in hospital is 70 days. But in Leicester the cases are not being kept in hospital for half that length of time, the average being only 34·5 days. It is clear, then, that if in London the patients were kept in no longer than they are in Leicester, there would be plenty of beds and to spare for every case that asks for admission. The answer which will be at once made to this is that at Leicester the patients are sent out while still in an infectious condition, as is shown by the 3 per cent. of return cases. But even London has not been absolutely free from complaints in regard to this, notwithstanding the length of time the patients have been kept in, and the question is whether it is worth while keeping the patients in the hospitals so long on the chance of saving this odd 3 per cent. of return cases, if so doing involves leaving scores and scores of other cases entirely unisolated during the whole period of their active infectiousness. We do not say that the Asylums Board managers are wrong. Their duty is towards their patients; no duty towards London has ever been imposed upon them beyond that of providing beds for such paupers as are suffering from infectious disease. But we do say that it is an absurd position that the sanitary authorities of London should possess no isolation hospitals, while the authority which does possess these hospitals is under no obligation to use them for the best advantage of the districts that pay the money for their support.

